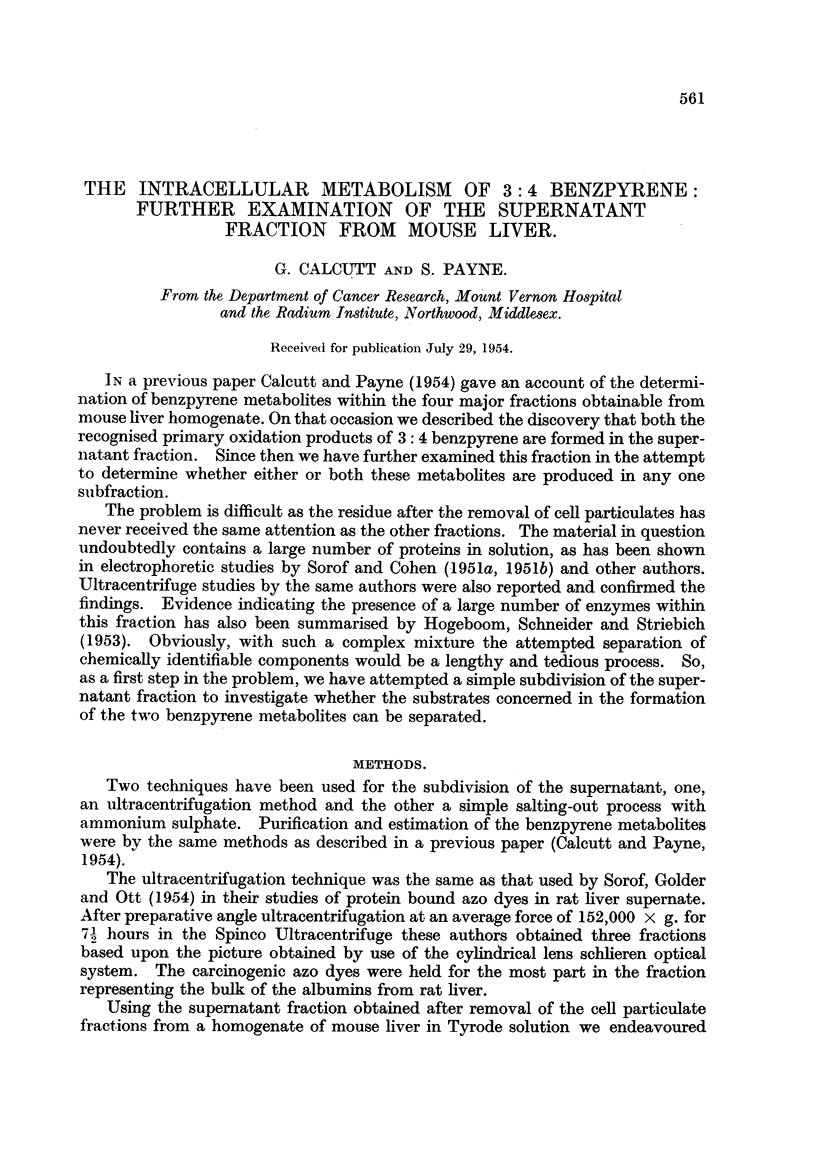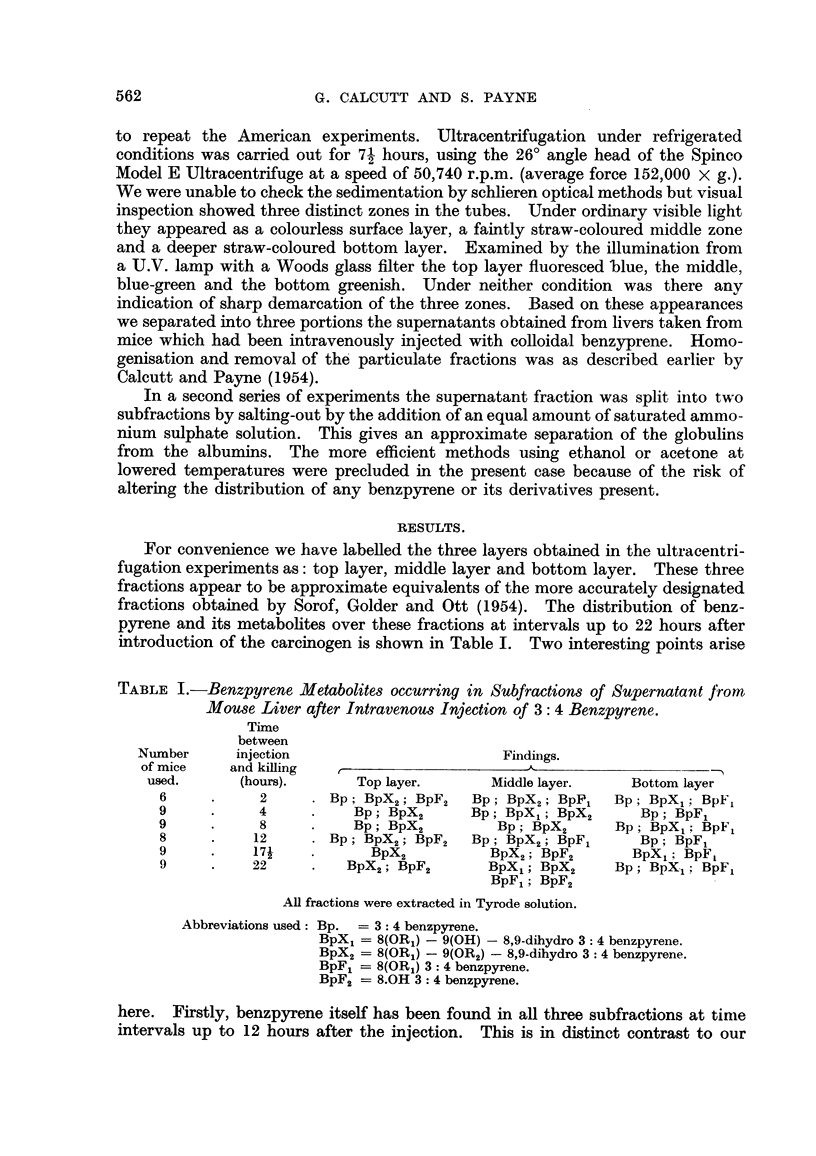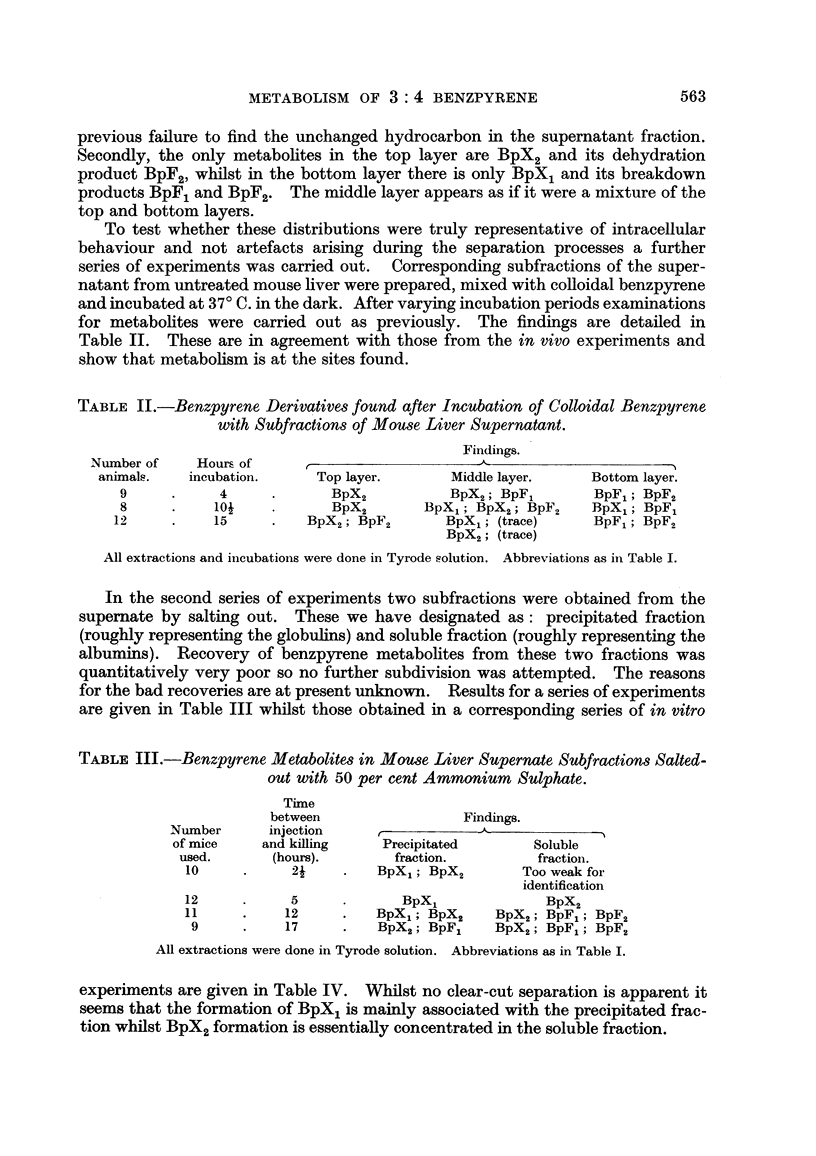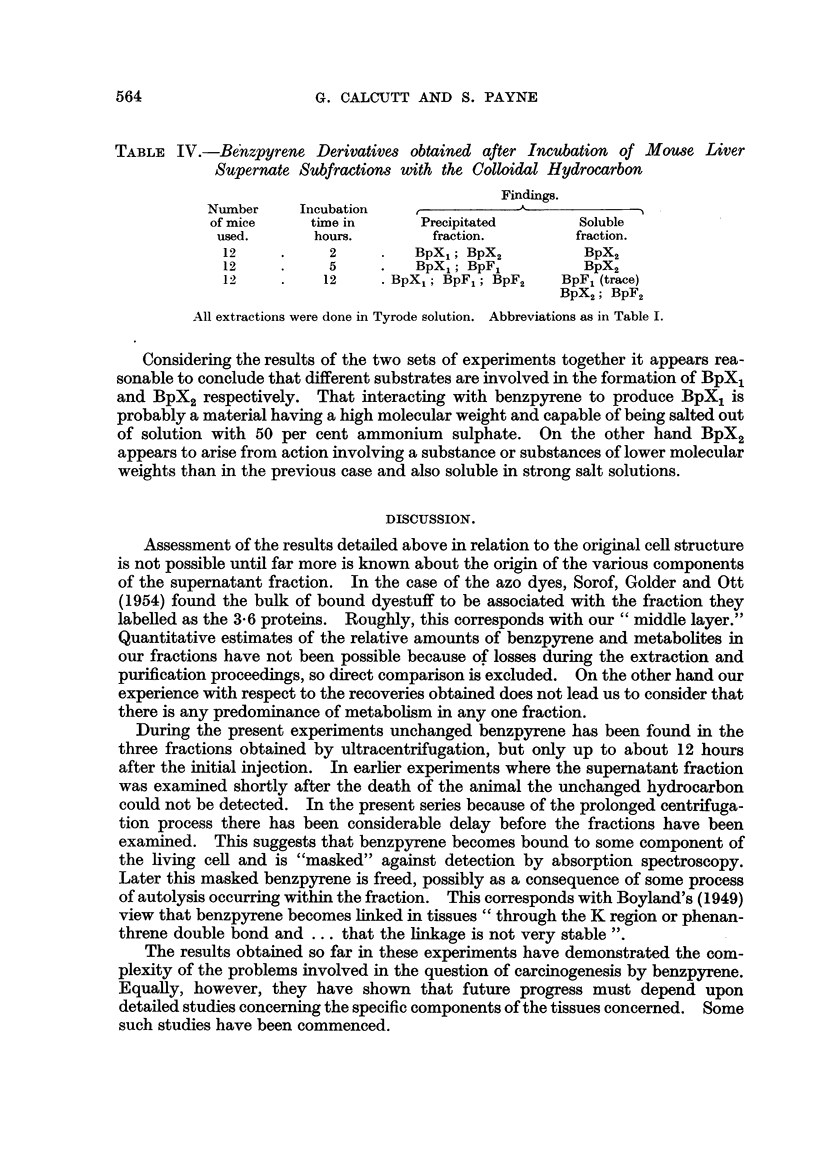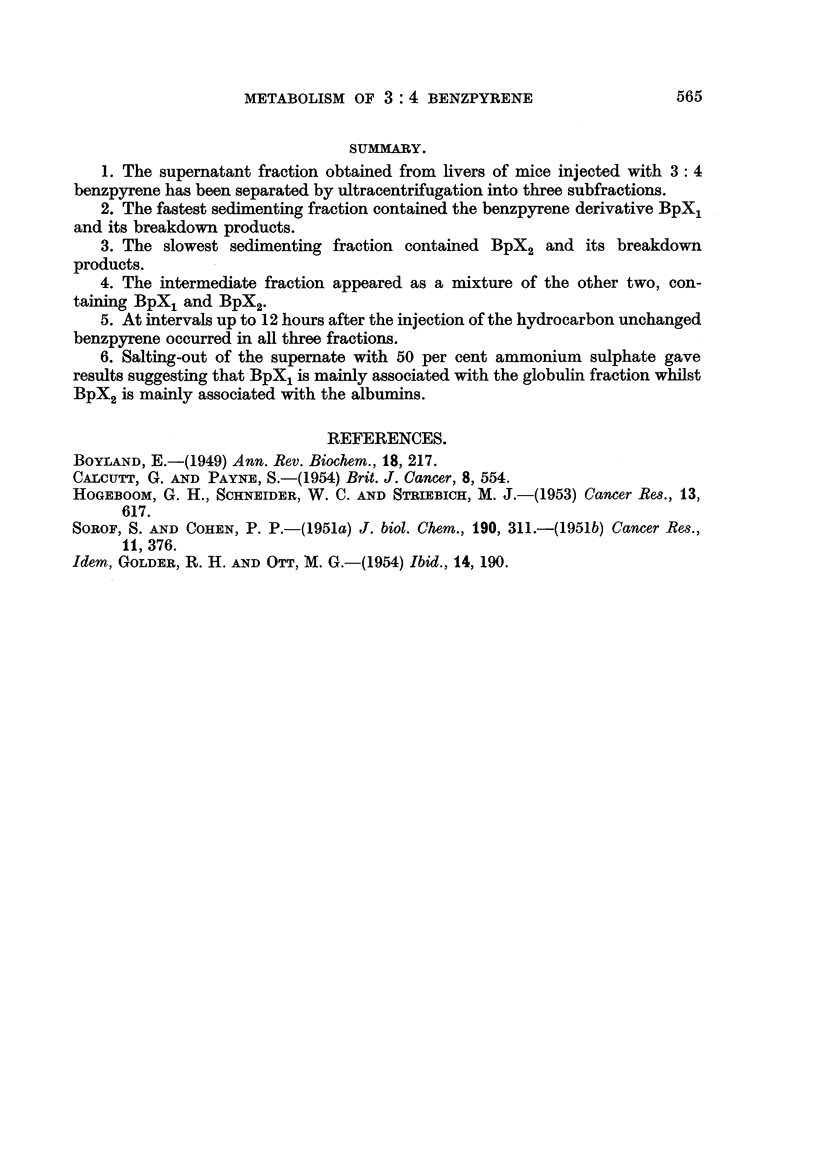# The Intracellular Metabolism of 3: 4 Benzypyrene: Further Examination of the Supernatant Fraction from Mouse Liver

**DOI:** 10.1038/bjc.1954.60

**Published:** 1954-09

**Authors:** G. Calcutt, S. Payne


### THE INTRACELLULAR METABOLISM OF 3 : 4 BENZPYRENE: FURTHER EXAMINATION OF THE SUPERNATANT FRACTION FROM MOUSE LIVER.

G. CALCUTT AND S. PAYNE.

*From the Department of Cancer Research, Mount Vernon Hospital and the Radium Institute, Northwood, Middlesex.*

Received for publication July 29, 1954.

IN a previous paper Calcutt and Payne (1954) gave an account of the determination of benzpyrene metabolites within the four major fractions obtainable from mouse liver homogenate. On that occasion we described the discovery that both the recognised primary oxidation products of 3 : 4 benzpyrene are formed in the supernatant fraction. Since then we have further examined this fraction in the attempt to determine whether either or both these metabolites are produced in any one subfraction.

The problem is difficult as the residue after the removal of cell particulates has never received the same attention as the other fractions. The material in question undoubtedly contains a large number of proteins in solution, as has been shown in electrophoretic studies by Sorof and Cohen (1951*a*, 1951*b*) and other authors. Ultracentrifuge studies by the same authors were also reported and confirmed the findings. Evidence indicating the presence of a large number of enzymes within this fraction has also been summarised by Hogeboom, Schneider and Striebich (1953). Obviously, with such a complex mixture the attempted separation of chemically identifiable components would be a lengthy and tedious process. So, as a first step in the problem, we have attempted a simple subdivision of the supernatant fraction to investigate whether the substrates concerned in the formation of the two benzpyrene metabolites can be separated.

### METHODS.

Two techniques have been used for the subdivision of the supernatant, one, an ultracentrifugation method and the other a simple salting-out process with ammonium sulphate. Purification and estimation of the benzpyrene metabolites were by the same methods as described in a previous paper (Calcutt and Payne, 1954).

The ultracentrifugation technique was the same as that used by Sorof, Golder and Ott (1954) in their studies of protein bound azo dyes in rat liver supernate. After preparative angle ultracentrifugation at an average force of 152,000 × g. for $7\frac{1}{2}$ hours in the Spinco Ultracentrifuge these authors obtained three fractions based upon the picture obtained by use of the cylindrical lens schlieren optical system. The carcinogenic azo dyes were held for the most part in the fraction representing the bulk of the albumins from rat liver.

Using the supernatant fraction obtained after removal of the cell particulate fractions from a homogenate of mouse liver in Tyrode solution we endeavoured to repeat the American experiments. Ultracentrifugation under refrigerated conditions was carried out for 7½ hours, using the 26° angle head of the Spinco Model E Ultracentrifuge at a speed of 50,740 r.p.m. (average force 152,000 × g.). We were unable to check the sedimentation by schlieren optical methods but visual inspection showed three distinct zones in the tubes. Under ordinary visible light they appeared as a colourless surface layer, a faintly straw-coloured middle zone and a deeper straw-coloured bottom layer. Examined by the illumination from a U.V. lamp with a Woods glass filter the top layer fluoresced blue, the middle, blue-green and the bottom greenish. Under neither condition was there any indication of sharp demarcation of the three zones. Based on these appearances we separated into three portions the supernatants obtained from livers taken from mice which had been intravenously injected with colloidal benzyprene. Homogenisation and removal of the particulate fractions was as described earlier by Calcutt and Payne (1954).

In a second series of experiments the supernatant fraction was split into two subfractions by salting-out by the addition of an equal amount of saturated ammonium sulphate solution. This gives an approximate separation of the globulins from the albumins. The more efficient methods using ethanol or acetone at lowered temperatures were precluded in the present case because of the risk of altering the distribution of any benzpyrene or its derivatives present.

### RESULTS.

For convenience we have labelled the three layers obtained in the ultracentrifugation experiments as: top layer, middle layer and bottom layer. These three fractions appear to be approximate equivalents of the more accurately designated fractions obtained by Sorof, Golder and Ott (1954). The distribution of benzpyrene and its metabolites over these fractions at intervals up to 22 hours after introduction of the carcinogen is shown in Table I. Two interesting points arise

TABLE I.—*Benzpyrene Metabolites occurring in Subfractions of Supernatant from Mouse Liver after Intravenous Injection of* 3 : 4 *Benzpyrene.*

| Number of mice used. | Time between injection and killing (hours). | Findings. | | |
|---|---|---|---|---|
| | | Top layer. | Middle layer. | Bottom layer |
| 6 | 2 | Bp ; $BpX_2$ ; $BpF_2$ | Bp ; $BpX_2$ ; $BpF_1$ | Bp ; $BpX_1$ ; $BpF_1$ |
| 9 | 4 | Bp ; $BpX_2$ | Bp ; $BpX_1$ ; $BpX_2$ | Bp ; $BpF_1$ |
| 9 | 8 | Bp ; $BpX_2$ | Bp ; $BpX_2$ | Bp ; $BpX_1$ ; $BpF_1$ |
| 8 | 12 | Bp ; $BpX_2$ ; $BpF_2$ | Bp ; $BpX_2$ ; $BpF_1$ | Bp ; $BpF_1$ |
| 9 | 17½ | $BpX_2$ | $BpX_2$ ; $BpF_2$ | $BpX_1$ ; $BpF_1$ |
| 9 | 22 | $BpX_2$ ; $BpF_2$ | $BpX_1$ ; $BpX_2$ $BpF_1$ ; $BpF_2$ | Bp ; $BpX_1$ ; $BpF_1$ |

All fractions were extracted in Tyrode solution.

Abbreviations used : Bp. = 3 : 4 benzpyrene.

$BpX_1$ = $8(OR_1) - 9(OH)$ — 8,9-dihydro 3 : 4 benzpyrene.

$BpX_2$ = $8(OR_1) - 9(OR_2)$ — 8,9-dihydro 3 : 4 benzpyrene.

$BpF_1$ = $8(OR_1)$ 3 : 4 benzpyrene.

$BpF_2$ = 8.OH 3 : 4 benzpyrene.

here. Firstly, benzpyrene itself has been found in all three subfractions at time intervals up to 12 hours after the injection. This is in distinct contrast to our previous failure to find the unchanged hydrocarbon in the supernatant fraction. Secondly, the only metabolites in the top layer are $BpX_2$ and its dehydration product $BpF_2$, whilst in the bottom layer there is only $BpX_1$ and its breakdown products $BpF_1$ and $BpF_2$. The middle layer appears as if it were a mixture of the top and bottom layers.

To test whether these distributions were truly representative of intracellular behaviour and not artefacts arising during the separation processes a further series of experiments was carried out. Corresponding subfractions of the supernatant from untreated mouse liver were prepared, mixed with colloidal benzpyrene and incubated at 37° C. in the dark. After varying incubation periods examinations for metabolites were carried out as previously. The findings are detailed in Table II. These are in agreement with those from the *in vivo* experiments and show that metabolism is at the sites found.

TABLE II.—*Benzpyrene Derivatives found after Incubation of Colloidal Benzpyrene with Subfractions of Mouse Liver Supernatant.*

| Number of animals. | Hours of incubation. | Findings. Top layer. | Middle layer. | Bottom layer. |
|---|---|---|---|---|
| 9 | 4 | $BpX_2$ | $BpX_2$ ; $BpF_1$ | $BpF_1$ ; $BpF_2$ |
| 8 | 10½ | $BpX_2$ | $BpX_1$ ; $BpX_2$ ; $BpF_2$ | $BpX_1$ ; $BpF_1$ |
| 12 | 15 | $BpX_2$ ; $BpF_2$ | $BpX_1$ ; (trace) $BpX_2$ ; (trace) | $BpF_1$ ; $BpF_2$ |

All extractions and incubations were done in Tyrode solution. Abbreviations as in Table I.

In the second series of experiments two subfractions were obtained from the supernate by salting out. These we have designated as : precipitated fraction (roughly representing the globulins) and soluble fraction (roughly representing the albumins). Recovery of benzpyrene metabolites from these two fractions was quantitatively very poor so no further subdivision was attempted. The reasons for the bad recoveries are at present unknown. Results for a series of experiments are given in Table III whilst those obtained in a corresponding series of *in vitro*

TABLE III.—*Benzpyrene Metabolites in Mouse Liver Supernate Subfractions Salted-out with* 50 *per cent Ammonium Sulphate.*

| Number of mice used. | Time between injection and killing (hours). | Findings. Precipitated fraction. | Soluble fraction. |
|---|---|---|---|
| 10 | 2½ | $BpX_1$ ; $BpX_2$ | Too weak for identification |
| 12 | 5 | $BpX_1$ | $BpX_2$ |
| 11 | 12 | $BpX_1$ ; $BpX_2$ | $BpX_2$ ; $BpF_1$ ; $BpF_2$ |
| 9 | 17 | $BpX_2$ ; $BpF_1$ | $BpX_2$ ; $BpF_1$ ; $BpF_2$ |

All extractions were done in Tyrode solution. Abbreviations as in Table I.

experiments are given in Table IV. Whilst no clear-cut separation is apparent it seems that the formation of $BpX_1$ is mainly associated with the precipitated fraction whilst $BpX_2$ formation is essentially concentrated in the soluble fraction.

TABLE IV.—*Benzpyrene Derivatives obtained after Incubation of Mouse Liver Supernate Subfractions with the Colloidal Hydrocarbon*

| Number of mice used. | Incubation time in hours. | Findings. Precipitated fraction. | Findings. Soluble fraction. |
|---|---|---|---|
| 12 | 2 | $BpX_1$ ; $BpX_2$ | $BpX_2$ |
| 12 | 5 | $BpX_1$ ; $BpF_1$ | $BpX_2$ |
| 12 | 12 | $BpX_1$ ; $BpF_1$ ; $BpF_2$ | $BpF_1$ (trace) $BpX_2$ ; $BpF_2$ |

All extractions were done in Tyrode solution. Abbreviations as in Table I.

Considering the results of the two sets of experiments together it appears reasonable to conclude that different substrates are involved in the formation of $BpX_1$ and $BpX_2$ respectively. That interacting with benzpyrene to produce $BpX_1$ is probably a material having a high molecular weight and capable of being salted out of solution with 50 per cent ammonium sulphate. On the other hand $BpX_2$ appears to arise from action involving a substance or substances of lower molecular weights than in the previous case and also soluble in strong salt solutions.

### DISCUSSION.

Assessment of the results detailed above in relation to the original cell structure is not possible until far more is known about the origin of the various components of the supernatant fraction. In the case of the azo dyes, Sorof, Golder and Ott (1954) found the bulk of bound dyestuff to be associated with the fraction they labelled as the 3·6 proteins. Roughly, this corresponds with our " middle layer." Quantitative estimates of the relative amounts of benzpyrene and metabolites in our fractions have not been possible because of losses during the extraction and purification proceedings, so direct comparison is excluded. On the other hand our experience with respect to the recoveries obtained does not lead us to consider that there is any predominance of metabolism in any one fraction.

During the present experiments unchanged benzpyrene has been found in the three fractions obtained by ultracentrifugation, but only up to about 12 hours after the initial injection. In earlier experiments where the supernatant fraction was examined shortly after the death of the animal the unchanged hydrocarbon could not be detected. In the present series because of the prolonged centrifugation process there has been considerable delay before the fractions have been examined. This suggests that benzpyrene becomes bound to some component of the living cell and is "masked" against detection by absorption spectroscopy. Later this masked benzpyrene is freed, possibly as a consequence of some process of autolysis occurring within the fraction. This corresponds with Boyland's (1949) view that benzpyrene becomes linked in tissues " through the K region or phenanthrene double bond and . . . that the linkage is not very stable ".

The results obtained so far in these experiments have demonstrated the complexity of the problems involved in the question of carcinogenesis by benzpyrene. Equally, however, they have shown that future progress must depend upon detailed studies concerning the specific components of the tissues concerned. Some such studies have been commenced.

SUMMARY.

1. The supernatant fraction obtained from livers of mice injected with 3 : 4 benzpyrene has been separated by ultracentrifugation into three subfractions.

2. The fastest sedimenting fraction contained the benzpyrene derivative $BpX_1$ and its breakdown products.

3. The slowest sedimenting fraction contained $BpX_2$ and its breakdown products.

4. The intermediate fraction appeared as a mixture of the other two, containing $BpX_1$ and $BpX_2$.

5. At intervals up to 12 hours after the injection of the hydrocarbon unchanged benzpyrene occurred in all three fractions.

6. Salting-out of the supernate with 50 per cent ammonium sulphate gave results suggesting that $BpX_1$ is mainly associated with the globulin fraction whilst $BpX_2$ is mainly associated with the albumins.

### REFERENCES.

Boyland, E.—(1949) *Ann. Rev. Biochem.*, **18**, 217.

Calcutt, G. and Payne, S.—(1954) *Brit. J. Cancer*, **8**, 554.

Hogeboom, G. H., Schneider, W. C. and Striebich, M. J.—(1953) *Cancer Res.*, **13**, 617.

Sorof, S. and Cohen, P. P.—(1951*a*) *J. biol. Chem.*, **190**, 311.—(1951*b*) *Cancer Res.*, **11**, 376.

*Idem*, Golder, R. H. and Ott, M. G.—(1954) *Ibid.*, **14**, 190.